# A Chinese Herbal Formula to Improve General Psychological Status in Posttraumatic Stress Disorder: A Randomized Placebo-Controlled Trial on Sichuan Earthquake Survivors

**DOI:** 10.1155/2012/691258

**Published:** 2011-10-19

**Authors:** Xian-Ze Meng, Feng Wu, Pin-Kang Wei, Li-Juan Xiu, Jun Shi, Bin Pang, Da-Zhi Sun, Zhi-Feng Qin, Yi Huang, Lixing Lao

**Affiliations:** ^1^Department of Traditional Chinese Medicine, Shanghai Changzheng Hospital, Second Military Medical University, Shanghai 200001, China; ^2^Department of Internal Medicine, Air Force Sanatorium, Dujiangyan, Sichuan Province 611833, China; ^3^Department of Mathematics and Statistics, University of Maryland Baltimore County, 1000 Hilltop Circle, Baltimore, MD 21250, USA; ^4^Center for Integrative Medicine, University of Maryland School of Medicine, Baltimore, MD 21201, USA

## Abstract

*Introduction*. Posttraumatic stress disorder (PTSD) is accompanied by poor general psychological status (GPS). In the present study, we investigated the effects of a Chinese herbal formula on GPS in earthquake survivors with PTSD. *Methods*. A randomized, double-blind, placebo-controlled trial compared a Chinese herbal formula, *Xiao-Tan-Jie-Yu-Fang* (XTJYF), to placebo in 2008 Sichuan earthquake survivors with PTSD. Patients were randomized into XTJYF (*n* = 123) and placebo (*n* = 122) groups. Baseline-to-end-point score changes in the three global indices of the Symptom Checklist-90-Revised (SCL-90-R) and rates of response in the SCL global severity index (GSI) were the primary endpoints. A subanalysis of the nine SCL factors and the sleep quality score were secondary endpoints. *Results and Discussion*. Compared to placebo, the XTJYF group was significantly improved in all three SCL global indices (*P* = 0.001~0.028). More patients in the XTJYF group reported “much improved” than the placebo group (*P* = 0.001). The XTJYF group performed significantly better than control in five out of nine SCL factors (somatization, obsessive-compulsive behavior, depression, anxiety, and hostility (*P* = 0.001~0.036)), and in sleep quality score (*P* < 0.001). XTJYF produced no serious adverse events. These findings suggest that XTJYF may be an effective and safe treatment option for improving GPS in patients with PTSD.

## 1. Introduction

On May 12, 2008, an earthquake measuring 8.0 on the Richter scale hit Sichuan Province in southwestern China. According to the official data, more than 69,200 people were confirmed dead, more than 374,600 were seriously injured [1], and at least 5 million were left homeless [2]. Recent literature shows that posttraumatic stress disorder (PTSD) and other psychological disorders such as anxiety and depression were fairly common and highly comorbid in 2008 Sichuan earthquake survivors [3].

Posttraumatic stress disorder (PTSD) is a significant public health problem [4]. About 6.8% of adults develop PTSD in their lifetimes; 3.5% have the condition in any given year [5, 6]. Approximately 10%–50% of the survivors of traumatic events such as earthquakes and tsunamis will develop chronic PTSD [7], which often persists for years if untreated [8–10]. The disorder is characterized by flashbacks and avoidance or numbness as well as hyperarousal after experiencing, witnessing, or confronting actual or potential death, serious physical injury, or a threat to physical integrity [11]. In addition to these symptoms, co-morbid psychiatric disorders are extremely common. In the National Comorbidity Survey (USA), approximately 80% of individuals with PTSD also met criteria for at least one other disorder listed in the diagnostic and statistical manual of mental disorders-III (DSM-III) [4]. Patients with PTSD often manifest other complications such as depression, anxiety, obsessive-compulsive behavior, hostility, and paranoid ideation disorders [3, 12–16]. Co-morbid psychiatric disorders and related subclinical symptoms combined with core PTSD symptoms result in poor general psychological status (GPS). 

Selective serotonin reuptake inhibitors are the usual first level pharmacological treatment for PTSD [17–22]. Other lines of drugs, such as benzodiazepines and monoamine oxidase inhibitors, are also commonly used [23]. However, the effects of these pharmaceuticals are not always satisfactory [23–25], and undesirable side effects such as sleep disturbance, sexual dysfunction, and dizziness have been reported [23, 26–29]. 

 For centuries, traditional Chinese medicine (TCM) has been widely used in China and some other Asian countries for psychological disorders, and many classic herbal formulas have been used to treat such maladies [30–38]. *Xiao-Yao-San* is one of the most popular [30–36]. We developed a modified, granulated form of *Xiao-Yao-San, Xiao-Tan-Jie-Yu-Fang* (XTJYF), by adding additional herbs, mainly from another classic TCM formula* Er-Chen-Tang* for treating depression, and we studied the safety and effects of this modification in cancer patients with depression (see Table 1) [39]. Because we found the formula effective and observed no serious side effects, we hypothesized that XTJYF would improve GPS in PTSD patients. 

## 2. Subjects and Methods

### 2.1. Study Design and Setting

**Figure 1:**
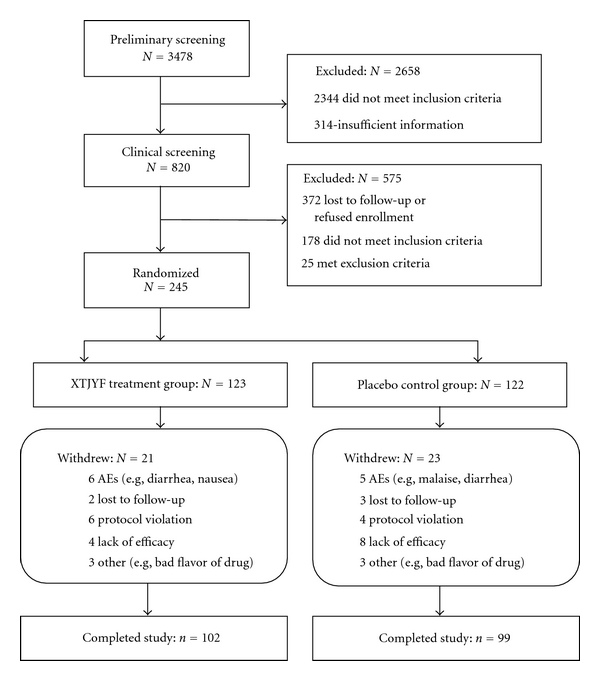
Flow chart of the study sample.

Patients were enrolled into this study five months after the 2008 Sichuan earthquake, between October 2008 and January 2009, through a community-based epidemiological survey of four settlements of a severely affected city, Dujiangyan. In the enrollment survey, the relationship between exposure to the earthquake and PTSD was assessed. Preliminary screening was performed in the communities by our researchers according to the DSM III for PTSD, Chinese version [40]. Eligible subjects were invited to participate in a diagnostic face-to-face or telephone interview with one of three experienced psychiatrists, each of which has at least eight years of clinical experience. Patients who met the inclusion and exclusion criteria were enrolled (see Patient Flow Chart, Figure 1), and our psychologists verified PTSD as the primary diagnosis of each enrollee. Inclusion criteria were age 16 or older, meeting DSM III criteria for PTSD with at least one of the nine Symptom Check-List-90-Revised (SCL-90-R) [41] subscores above the Chinese norm [42], and being willing to be randomly assigned. Participants understood that those randomized into the placebo control group could receive XTJYF after completion of the whole trial if they wished. Exclusion criteria were past history of bipolarism, schizophrenia, or other psychotic disorders; current organic mental disorder, factitious disorder, or malingering; any past history of alcohol or substance dependence or abuse; evidence of clinically significant hepatic or renal disease or any other acute or unstable medical condition that might interfere with safe participation in the study; use of any medication with clinically significant psychotropic activity within two weeks of randomization; any cognitive-behavioral therapy during the trial; psychotherapy initiated or ending during the trial. For female patients of childbearing age, participation was contingent on a negative serum pregnancy test and a medically accepted method of contraception.

Written informed consent was obtained from all patients before participation. Patients were free to withdraw from the study at any time. Clinical diagnoses, physicals, and laboratory examinations were mainly conducted in the outpatient clinic at the Air Force Sanatorium in the city of Dujiangyan by our psychologist and other investigators. The research staff collected patients' weekly feedback on their medical conditions and delivered the XTJYF or placebo through in-house visits. The trial protocol was approved by the Ethics Committee of Shanghai Changzheng Hospital and the Air Force Sanatorium in Dujiangyan.

A sociodemographic inventory and a medical history were taken, and a routine physical and laboratory examination (i.e., blood pressure, ECG, clinical chemistry and hematology tests, and urinalysis) was performed by the investigators as a baseline for future toxicology screening.

### 2.2. Randomization and Blinding

Eligible patients were randomized to either XTJYF treatment or placebo control. Random numbers were generated by computer software; treatment codes were held by the chief investigator, who was isolated from patients and outcome data. The chief investigator was also responsible for distributing the XTJYF and placebo with the assistance of our research staff. Patients, research staff, and data entry clerks were blinded to treatment group assignment. Treatment compliance was assessed by package count and observation by the research staff. Treatment codes were disclosed after the entire study was completed.

### 2.3. Study Interventions

All patients received 12 g packages of granulated XTJYF or placebo twice a day for eight weeks [39] and were instructed to drink the contents dissolved in warm, boiled water.

### 2.4. Outcome Measures

Each patient completed the SCL-90-R questionnaires twice, at baseline prior to randomization and in the eighth week after the randomization, that is, at the end of this clinical trial. The SCL-90-R is a questionnaire for self-reporting psychological distress. It is widely used in patients suffering from mental diseases and for psychological evaluation of healthy individuals. The instrument is well accepted for its good internal consistency, dimensional structure, reliability, and validity [43–45]. The Chinese SCL-90, translated and validated by Wang from the English version of the SCL-90-R, was used [46, 47].

The SCL-90-R consists of 90 symptoms of distress. Patients were instructed to indicate the degree to which they had been troubled by each symptom during the preceding week by ranking the symptom from 0 to 4, with 0 being “not at all” and 4 being “extremely.” The statements were classified into nine dimensions, or factors (F), that reflect various types of psychopathology: (F1) somatization, (F2) obsessive-compulsive behavior, (F3) interpersonal sensitivity, (F4) depression, (F5) anxiety, (F6) hostility, (F7) phobic anxiety, (F8) paranoid ideation, and (F9) psychoticism. Three supplementary global indices reflect the degree of symptomatology. The global severity index (GSI) registers the average depth of impairment based on the severity recorded for each symptom; the positive symptom total index (PST) indicates the total number of symptoms experienced; the Positive Symptom Distress Index (PSDI) reflects the level of distress by correlating the reported symptoms [41]. In addition, on the SCL-90-R, there are seven items not included in any of the nine factors, among which, three reflect sleep quality. Individual SCL-90-R factors have been used to evaluate the psychological condition of PTSD patients, and there is sufficient evidence to support the correlation of higher global SCL-90-R scores with the severity of a patient's core PTSD symptoms [12, 48–57].

During the trial, patients were closely monitored for adverse events (AEs) and worsening of symptoms. The time of onset of any observed or spontaneously reported AE, its duration and severity, any action taken, and the outcome were recorded.

### 2.5. Herbal Preparation and Dispensing

The original formula, *Xiao-Yao-San,* contains eight herbs: *Chai-Hu* (*Radix Bupleuri*), *Dang-Gui* (*Radix Angelicae sinensis*), *Fu-Ling* (*Poria*), *Bai-Zhu* (*Rhizoma Atractylodis macrocephalae*), *Bai-Shao* (*Radix Paeoniae alba*), *Bo-He* (*Herba Menthae*), *Zhi Gan-Cao *(*Radix Glycyrrhizae preparata*), and *Sheng-Jiang* (*Rhizoma Zingiberis recens*). Our modification, XTJYF, contains all the herbs of the original formula, except *Sheng-Jiang,* plus additional seven herbs, including *Fa Ban-Xia* (*Rhizoma Pinelliae preparatae*) and *Chen-Pi* (*Pericarpium Citri reticulatae*), that are commonly used for psychological disorders (see Table 1).

All herbal substances used in this trial are listed with the Pharmacopoeia Commission of China, 2005, and are accepted as suitable for human consumption when administered within standard dosage levels. None of these herbs is a controlled substance or an endangered species. Raw herbs were purchased from the Lei Yun Shang Pharmaceutical Company (Shanghai, China). The herbs were extracted with water, and the resulting granules were packaged by the Chinese Drug Preparation Department of Shanghai Changzheng Hospital. Levels of heavy metals and microbial and pesticide residues were carefully assessed, and all fell well within the normal range [58].

 The placebo granules, purchased from Jiangsu Tianjiang Pharmaceutical Company, Ltd., were designed to resemble the XTJYF granules in taste, smell, and appearance. The placebo was composed of dextrin, sunset yellow fcf, and a sweetener; the proportion was 1200 : 1 : 7. After being tested on five independent volunteers, the placebo was deemed indistinguishable from XTJYF. XTJYF and the placebo were dispensed in identical opaque packages.

### 2.6. Statistical Analysis

Quantitative data was summarized using mean, standard deviation (SD), or 95% confidence interval (95% CI). Qualitative data was described using proportion, as percentages. Baseline characteristics of the two groups were compared using the two-sided chi-square test or *t*-test at a significance level of 0.05. 

Since this was a randomized, blind clinical trial, the statistical analyses for treatment effect evaluation of the primary and secondary outcomes are relatively straightforward. Baseline-to-end-point score changes in the three global SCL-90-R indices and rates of response in the GSI were computed as the primary endpoints. For defining rate of response, patients with a reduction of at least 30% from the baseline GSI score were classified as “much improved”; at least 50%, as “very much improved.” Subanalyses of the baseline-to-end-point score changes of the nine SCL factors and sleep quality score (the average of the scores of the three SCl-90-R items on sleep quality) were secondary endpoints. Statistical analysis on both primary and secondary outcomes was done using intention-to-treat analysis (ITT) with statistical software SPSS. Missing values in the SCL-90-R questionnaire for the patients who withdrew from the study before the eighth week were imputed using the last-observation-carried-forward method. For primary outcomes, effect sizes (for three global indices) and number needed to treat (NNT, for rate of response in the GSI), as well as the *P* values from two sample *t*-tests and chi-square tests, are reported in the treatment effect assessment. The same analytic approaches were applied to the secondary outcomes. Additionally, Fisher's exact test was used to compare the difference in dropout rate and AEs between the two treatment groups.

## 3. Results

A total of 3478 individuals were screened, of whom 820 passed the preliminary screening and 245 were finally enrolled into the study; 575 were excluded. Of these, 372 were lost to follow-up or refused enrollment; 178 did not meet the inclusion criteria; 25 met the exclusion criteria. Enrolled patients were randomly assigned to XTJYF (*n* = 123) or placebo (*n* = 122) treatment. Of these, 102 (83%) of the XTJYF group and 99 (81%) of the control group completed the whole study. Reasons for withdrawal from the study are listed separately for each treatment group in Figure 1, and a detailed discussion on treatment tolerability is provided in Section 3.4.

### 3.1. Baseline Characteristics and GPS Assessment

Table 2 shows that randomization was effective and that there were no significant differences between the two groups in baseline demographics, core clinical PTSD symptoms, or baseline SCL-90-R global indices. Even though individual SCL-90-R factor scores and sleep quality scores at baseline are not shown here, we checked all of them and founded no significant differences between the two groups. Notice that women constituted 72% of XTJYF-treated and 71% of placebo-treated patients. Ages ranged from 16 to 85; 64% were over 45.

Table 3 shows the urgency of the public health needs of these earthquake-affected PTSD patients and indicates that baseline scores of the patients in our clinical trial are significantly higher in all nine SCL-90-R factors and all three supplementary global indices than those seen in Chinese and American norms [41, 42].

### 3.2. Treatment Effect on Primary Outcomes

**Figure 2:**
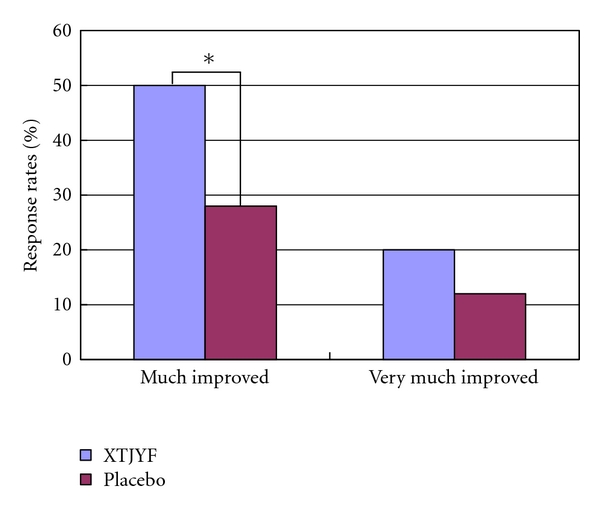
Treatment response rates of XTJYF versus placebo. Patients with a score reduction of at least 30% from the baseline SCL-90-R GSI score were classified as “much improved,” and 50%, as “very much improved.” *XTJYF versus placebo, *P* < 0.05.

Table 4 shows that patients in the XTJYF group experienced statistically significant improvement after treatment in all three supplementary global index scores compared to the placebo group. Based on the reported effect sizes, XTJYF treatment has a moderate effect on GSI and PSDI indices and a small effect on the PST index. Our findings on the rate of response, defined based on GSI score improvement, are displayed in Figure 2; 50% of the XTJYF patients versus 28% of those in the placebo group were “much improved,” providing statistically significant evidence supporting the advantage of XTJYF over placebo at the level of 0.05 (*P* value = 0.001). The NNT is 4.55. Additionally, as Figure 2 shows, 20% of the XTJYF patients versus 12% of those in the placebo group were “very much improved,” but this result is not statistically significant (*P* value = 0.12).

### 3.3. Treatment Effect on Secondary Outcomes

The second part of Table 4 displays the treatment effects of XTJYF and placebo on the nine SCL factors and sleep quality score. The results indicate that, in comparison to placebo, the XTJYF group experienced statistically significant improvement after treatment in five of the nine SCL factors, somatization (*P* = 0.003), obsessive-compulsive behavior (*P* = 0.036), depression (*P* = 0.001), anxiety (*P* < 0.001), and hostility (*P* = 0.019). Based on the reported effect sizes, XTJYF treatment has a moderate effect on somatization, depression, anxiety, and hostility, as well as a small effect on obsessive-compulsive behavior, interpersonal sensitivity, and phobic anxiety. Table 4 also shows that XTJYF treatment yielded statistically significant improvement in sleep quality at the end of the study, with a *P* value of less than 0.001 and a moderate effect size.

### 3.4. Treatment Tolerability

Overall, XTJYF was well tolerated. Compliance rate, 83% for the XTJYF and 81% for the placebo group, was reasonably high. Six in the XTJYF and five in the control group withdrew due to adverse effects, so reported AEs were similar in the two groups. The most frequently reported AEs were nausea (14.6% versus 9.0%; *P* = 0.24), diarrhea (10.6% versus 6.5%; *P* = 0.36), and malaise (10.6% versus 12.3%; *P* = 0.69). All AEs were minor and were determined to be unrelated to the ingestion of XTJYF. In the XTJYF group, 21 subjects dropped out (17.1%); in the placebo group, 23 did (18.9%, *P* = 0.74). The primary reasons cited for dropout in the XTJYF and placebo groups, respectively, were AE (4.9% versus 4.1%; *P* = 1); lost to follow-up (1.6% versus 2.5%; *P* = 0.68); protocol violation (4.9% versus 3.3%; *P* = 0.75); lack of efficacy (3.3% versus 6.6%; *P* = 0.25); miscellaneous reasons, for example, disliked the taste of the herbs (2.4% versus 2.5%; *P* = 1). Subjects' laboratory values and vital signs were similar in the two groups. Changes in these values were minor, infrequent, and not considered clinically meaningful.

## 4. Discussion

In the present study, we compared our data to the Chinese norm calculated by Jin et al. [42] and to the USA norm published by Derogatis [41]. (see Table 3). At baseline, the nine SCL factors and three global indices were higher than the norm in these earthquake-related PTSD subjects, suggesting that earthquake-related PTSD is accompanied by poor GPS. These findings are consistent with those reported by other investigators [3, 59–63].

Hypothesizing that it would improve poor GPS in earthquake-related PTSD, we investigated a Chinese herbal formula, XTJYF, modified from a classic formula, *Xiao-Yao-San*, and found that, compared to placebo, XTJYF significantly improved all of the three global indices of SCl-90-R, and a significantly greater proportion of patients were “much improved” according to changes in GSI score. (See Table 4 and Figure 2). A subanalysis provided a more detailed look at specific XTJYF effects on poor GPS, showing that five of the nine SCL factors and sleep quality score improved. (see Table 4).

These findings suggest that XTJYF may globally improve GPS in earthquake-related PTSD patients, specifically in somatization, obsessive-compulsive behavior, depression, anxiety, and hostility. In addition, the formula may improve the sleep quality of the patients and appears to be safe. Although a few subjects reported gastrointestinal complaints such as nausea and diarrhea during treatment, these were probably due to the poor diet available after the earthquake; these symptoms were also frequently reported in the placebo control group. Our findings are consistent with those of our previous study on XTJYF for cancer patients with depression [39].

The results are meaningful because all five of the psychological disorders mentioned above are associated with high levels of functional and psychosocial disability in chronic PTSD patients [3, 4, 6, 12–16, 64–69], and most are reported to predict greater refractoriness to routine therapy in individuals diagnosed with PTSD [17, 70–73]. For example, PTSD patients who report somatic symptoms also report higher overall PTSD symptoms [15, 64] and a higher frequency of depression [64, 74]. Patients with co-morbid PTSD and obsessive-compulsive behavior have been found to have a poorer response to cognitive behavioral therapy than those diagnosed with PTSD alone [73]. Co-morbid PTSD/depression appears to predict greater refractoriness to pharmacotherapy, greater symptom severity, lower levels of functioning and rates of recovery, and increased disability and potential of suicide [4, 6, 65–67]. Like depression, anxiety symptoms are associated with lower quality-of-life estimates and greater refractoriness to routine pharmacotherapy in PTSD patients [3, 68]. Hostility, which according to a meta-analysis of 39 studies is significantly elevated in individuals with PTSD [16], is linked to adverse health outcomes, including cardiac death [69]. In the present study, XTJYF also appears to improve the sleep quality of these PTSD patients; sleep disturbances are among the most treatment-resistant symptoms of PTSD [75]. All of these symptoms are likely to contribute to alcohol and drug abuse [76, 77] as well as suicidal ideation [78]. 

The psychological mechanisms of action of *Xiao-Yao-San* and its modifications have been investigated. It has been reported that the formula may act on psychological symptoms by upregulating central neurotransmitters such as serotonin. Bao et al. [79] reported that *Xiao-Yao-San* produced antidepressant effects in a mouse model of depression by ameliorating brain cortex 5-HT and 5-HIAA content. Other mechanisms of the formula have also been reported. Yue et al. [80] reported that *Xiao-Yao-San *suppressed chronic stress in a rat model by up-regulating GluR2/3, the AMPA receptor subunit 2/3, which mediates the postsynaptic depolarization that initiates neuronal firing [81], and by downregulating PICK1, a protein that interacts with C-kinase 1, which may lead to AMPA receptor anchorage [82] in hippocampal regions CA1 and CA3. Similar findings, that *Xiao-Yao-San* upregulates AMPA receptor subunit mRNA expression in hippocampal region CA1 and the amygdala, were reported [83]. Furthermore, *Xiao-Yao-San *and its modifications were reported to suppress chronic stress by maintaining the stability of hippocampal neurons [84], inhibiting hypothalamic-pituitary-adrenocortical axis negative feedback regulation [85], and counteracting increase of Ca^2+^ concentration in hippocampal synaptosomes [86]. Based on TCM theory, seven drugs were added in our modification, mainly from another classic TCM formula, *Er-Chen-Tang*. According to our previous preclinical study, this modification may suppress depression by up-regulating the 5-HT_1A_ receptor in the hippocampus in a rat model of chronic stress [87]. However, because *Xiao-Yao-San* and its modifications contain multiple ingredients, specific active ingredients have not been identified, and the herbal interactions within the formula have not been systematically investigated. Further investigation to elucidate the mechanisms of action of this formula is warranted.

Several limitations to this study should be noted. First, our trial lacked a long follow-up assessment. This was largely due to the difficulties in following up this particular population, which consisted of earthquake survivors living in shelters with no specific address. In the patient recruitment stage, more than 45% (372 of 820) of those preliminarily screened for PTSD were lost to follow-up. Secondly, we did not include a questionnaire measuring specific PTSD core symptoms, mainly because of the low level of education in this mountain population. In our patient population, 43% had an elementary education or less and found it difficult to complete a single 90-question SCL-90-R questionnaire. However, although we did not include a specific questionnaire such as the Clinician-Administered PTSD Scale [88] or the Clinician-Rated Treatment Outcome PTSD Scale [89] to measure core PTSD symptoms, the widely used SCL-90-R captures a broader patient psychological profile than a specific PTSD questionnaire would do. Thirdly, only one dosage of XTJYF was used in this study, that used in our standard clinical practice. A higher dosage might benefit the nonresponders. Finally, more detailed information on types of trauma and the percentages of patients who suffered them should be gathered and analyzed.

 Despite the limitations, our findings provide preliminary support for the use of TCM in treating GPS in earthquake survivors with PTSD. TCM has been used extensively in China to treat people suffering from various diseases after disasters, for it is readily available, reasonably cheap, effective, and safe. Because of their wide usage, the production of TCM herbal products is quick and cost effective in China. Traditional Chinese herbal medicine may provide an adjuvant therapy that is safe, effective, and timely for affected populations in natural disasters such as earthquakes.

## Figures and Tables

**Table 1:** Ingredients of Xiao-Tan-Jie-Yu-Fang.

No.	Chinese name	Pharmaceutical name	Proportion, %
(1)	Chai-Hu	*Radix Bupleuri*	4.5
(2)	Dang-Gui	*Radix Angelicae sinensis*	4.5
(3)	Fu-Ling	*Poria*	15.2
(4)	Chao Bai-Zhu	*Rhizoma Atractylodis macrocephalae *(parched)	4.5
(5)	Chao Bai-Shao	*Radix Paeonia alba* (parched)	7.6
(6)	Bo-He	*Herba Menthae *	3.0
(7)	Zhi Gan-Cao	*Radix Glycyrrhizae preparatae*	3.0
(8)	Huang-Lian	*Rhizoma Coptidis*	1.5
(9)	Fa Ban-Xia	*Rhizoma Pinelliae preparatae*	7.6
(10)	Chen-Pi	*Pericarpium Citri reticulatae*	4.5
(11)	Duan Long-Gu	*Os Draconis* (calcined)	15.0
(12)	Duan Mu-Li	*Concha Ostreae* (calcined)	15.0
(13)	Zhi Da-Huang	*Radix et Rhizoma Rhei preparatae*	6.1
(14)	Shi-Changpu	*Rhizoma Acori graminei*	7.6

**Table 2:** Baseline characteristics, earthquake-affected PTSD patient treatment groups.

Variable	XTJYF (*N *= 123)	Placebo (*N* = 122)	*P*
Sex, *n* (%)			
Female	88 (71.5)	86 (70.5)	0.89
Male	35 (28.5)	36 (29.5)	
Age, mean (SD)	51.2 (15.0)	51.0 (16.0)	0.93
Marital status, *n* (%)			
Married or living together	99 (80.5)	96 (78.7)	0.75
Others (unmarried, divorced, etc.)	24 (19.5)	26 (21.3)	
Education, *n* (%)			
Primary school or less	51 (41.5)	53 (43.4)	0.80
More than primary school	72 (58.5)	69 (56.6)	
Occupation, *n* (%)			
Farmer or unemployed	89 (72.4)	86 (70.5)	0.78
Other employment or retired	34 (27.6)	24 (19.5)	
Clinical PTSD symptom data, *n* (%)			
Uncontrollable recall of earthquake experiences	81 (65.9)	85 (69.7)	0.59
Repeated nightmares of earthquake	53 (43.1)	54 (44.3)	0.90
Repeated hallucinations	46 (37.4)	38 (31.1)	0.35
Heart racing, sweating, pallor when viewing earthquake ruins or victims	91 (74.0)	82 (67.2)	0.26
Poor sleep	87 (70.7)	86 (70.5)	1.00
Tense or easily agitated	97 (78.9)	92 (75.4)	0.55
Lack of concentration	60 (48.8)	50 (41.0)	0.25
Panic	52 (42.3)	45 (36.9)	0.43
Avoids recalling anything related to the earthquake	73 (59.3)	73 (59.8)	1.00
Avoids activities related to earthquake	56 (45.5)	55 (45.1)	1.00
Avoids contact with others, indifferent to relatives	54 (43.9)	45 (36.9)	0.30
Loss of interest and motivation	51 (41.5)	55 (45.1)	0.61
Selectively forgetful	67 (54.5)	62 (50.8)	0.61
Loss of hope for the future	34 (27.6)	39 (32.0)	0.49
Lost relatives in the earthquake, *n* (%)	7 (5.7)	11 (9.0)	0.34
Baseline outcome measures from SCL-90-R, mean (SD)			
Global severity index	1.14 (0.61)	1.12 (0.60)	0.78
Positive symptom total index	48.4 (20.0)	48.8 (18.8)	0.87
Positive symptom distress index	2.14 (0.97)	2.00 (0.52)	0.18

**Table 3:** SCL-90-R Factor scores, Chinese and American norms compared to earthquake-affected PTSD patients at baseline.

Sample	Norm, China^*⋆*^	Norm, USA^●^	PTSD, Sichuan
SCL-90-R	*N *= 1388	*N *= 974	*N *= 245
	Mean (95% CI)	Mean (95% CI)	Mean (95% CI)
Somatization	0.37 (0.34–0.40)	0.36 (0.33–0.39)	1.20 (1.11–1.29)*
Obsessive-compulsive behavior	0.62 (0.59–0.65)	0.39 (0.36–0.42)	1.38 (1.29–1.47)*
Interpersonal sensitivity	0.65 (0.62–0.68)	0.29 (0.27–0.31)	0.93 (0.84–1.02)*
Depression	0.50 (0.47–0.53)	0.36 (0.33–0.39)	1.29 (1.18–1.39)*
Anxiety	0.39 (0.37–0.41)	0.30 (0.28–0.32)	1.25 (1.15–1.34)*
Hostility	0.46 (0.43–0.49)	0.30 (0.27–0.33)	1.12 (1.02–1.23)*
Phobic anxiety	0.23 (0.21–0.25)	0.13 (0.11–0.15)	0.93 (0.83–1.03)*
Paranoid ideation	0.43 (0.40–0.46)	0.34 (0.31–0.37)	0.74 (0.65–0.83)*
Psychoticism	0.29 (0.27–0.31)	0.14 (0.12–0.16)	0.77 (0.69–0.85)*
Global severity index		0.31 (0.29–0.33)	1.13 (1.05–1.20)^∆^
Positive symptom total index	24.9 (24.0–25.9)	19.3 (18.3–20.3)	48.6 (46.2–51.1)*
Positive symptom distress index		1.32 (1.29–1.35)	2.07 (1.97–2.17)^∆^

^*⋆*^The original data was obtained from Jin et al. [42]. We recalculated the original data from “mean (sd)” to “mean (95% CI)” in order to make these data comparable. ^●^The original data was obtained from Derogatis [41]. We recalculated the original data from “mean (sd)” to “mean (95% CI)” in order to make these data comparable.*Compared to the Chinese and American norms, *P* < 0.05. ^∆^Compared to the American norms, *P* < 0.05.

**Table 4:** XTJYF treatment effect on primary and secondary outcomes.^(1)^

SCL-90-R factor	XTJYF (*N* = 123)	Placebo (*N* = 122)	Effect size^(2)^	*P* ^(3)^
	Mean (95% CI)	Mean (95% CI)		
*Primary outcomes *				
Global severity index	0.30 (0.24–0.37)	0.15 (0.09–0.21)	**0.424**	**0.001**
Positive symptom total index	6.66 (4.58–8.73)	3.52 (1.62–5.41)	0.284	**0.028**
Positive symptom distress index	0.38 (0.30–0.45)	0.19 (0.12–0.26)	**0.448**	**0.001**
*Secondary Outcomes*				
Somatization	0.34 (0.25–0.43)	0.16 (0.08–0.24)	**0.391**	**0.003**
Obsessive-compulsive behavior	0.28 (0.20–0.36)	0.15 (0.07–0.24)	0.270	**0.036**
Interpersonal sensitivity	0.27 (0.19–0.35)	0.16 (0.08–0.24)	0.241	0.061
Depression	0.35 (0.27–0.44)	0.16 (0.08–0.24)	**0.420**	**0.001**
Anxiety	0.40 (0.29–0.50)	0.12 (0.04–0.21)	**0.500**	**<0.001**
Hostility	0.31 (0.21–0.40)	0.15 (0.06–0.24)	**0.304**	**0.019**
Phobic anxiety	0.23 (0.14–0.32)	0.13 (0.05–0.21)	0.211	0.101
Paranoid ideation	0.16 (0.08–0.24)	0.12 (0.04–0.21)	0.070	0.586
Psychoticism	0.19 (0.11–0.26)	0.15 (0.08–0.23)	0.077	0.548
Sleep quality	0.76 (0.58–0.94)	0.33 (0.19–0.47)	**0.467**	**<0.001**

^(1)^Statistical analysis was done using intent-to-treat analysis (ITT) with SPSS. ^(2)^Cohen's d effect size measure, in which an effect size of 0.2 to 0.3 is considered a “small” effect, around 0.5, a “medium” effect, and 0.8 to infinity, a “large” effect, is used here. ^(3)^The *P* values come from the two sample *t*-tests.

## References

[1] http://www.scio.gov.cn/zxbd/wz/200905/t310218.htm.

[2] http://www.china.com.cn/news/2008-09/04/content_16386369.htm.

[3] Fan F, Zhang Y, Yang Y, Mo L, Liu X (2011). Symptoms of posttraumatic stress disorder, depression, and anxiety among adolescents following the 2008 Wenchuan earthquake in China. *Journal of Traumatic Stress*.

[4] Kessler RC, Sonnega A, Bromet E, Hughes M, Nelson CB (1995). Posttraumatic stress disorder in the national comorbidity survey. *Archives of General Psychiatry*.

[5] Kessler RC, Berglund P, Demler O, Jin R, Merikangas KR, Walters EE (2005). Lifetime prevalence and age-of-onset distributions of DSM-IV disorders in the national comorbidity survey replication. *Archives of General Psychiatry*.

[6] Kessler RC, Chiu WT, Demler O, Merikangas KR, Walters EE (2005). Prevalence, severity, and comorbidity of 12-month DSM-IV disorders in the national comorbidity survey replication. *Archives of General Psychiatry*.

[7] Neria Y, Nandi A, Galea S (2008). Post-traumatic stress disorder following disasters: a systematic review. *Psychological Medicine*.

[8] Bland SH, Valoroso L, Stranges S, Strazzullo P, Farinaro E, Trevisan M (2005). Long-term follow-up of psychological distress following earthquake experiences among working Italian males: a cross-sectional analysis. *Journal of Nervous and Mental Disease*.

[9] Chen CH, Tan HKL, Liao LR (2007). Long-term psychological outcome of 1999 Taiwan earthquake survivors: a survey of a high-risk sample with property damage. *Comprehensive Psychiatry*.

[10] Su CY, Tsai KY, Chou FHC, Ho WW, Liu R, Lin WK (2010). A three-year follow-up study of the psychosocial predictors of delayed and unresolved post-traumatic stress disorder in Taiwan Chi-Chi earthquake survivors. *Psychiatry and Clinical Neurosciences*.

[11] American Psychiatric and Association (2000). *Diagnostic and Statistical Manual of Mental Disorders-Text Revision (DSM-IV-TR)*.

[12] Huppert JD, Moser JS, Gershuny BS (2005). The relationship between obsessive-compulsive and posttraumatic stress symptoms in clinical and non-clinical samples. *Journal of Anxiety Disorders*.

[13] Keane TM, Kaloupek DG (1997). Comorbid psychiatric disorders in PTSD. Implications for research. *Annals of the New York Academy of Sciences*.

[14] Brady KT, Killeen TK, Brewerton T, Lucerini S (2000). Comorbidity of psychiatric disorders and posttraumatic stress disorder. *Journal of Clinical Psychiatry*.

[15] Brady KT (1997). Posttraumatic stress disorder and comorbidity: recognizing the many faces of PTSD. *Journal of Clinical Psychiatry*.

[16] Orth U, Wieland E (2006). Anger, hostility, and posttraumatic stress disorder in trauma-exposed adults: a meta-analysis. *Journal of Consulting and Clinical Psychology*.

[17] Marshall RD, Beebe KL, Oldham M, Zaninelli R (2001). Efficacy and safety of paroxetine treatment for chronic PTSD: a fixed-dose, placebo-controlled study. *American Journal of Psychiatry*.

[18] Brady K, Pearlstein T, Asnis GM (2000). Efficacy and safety of sertraline treatment of posttraumatic stress disorder: a randomized controlled trial. *Journal of the American Medical Association*.

[19] Davidson JRT, Rothbaum BO, Van der Kolk BA, Sikes CR, Farfel GM (2001). Multicenter, double-blind comparison of sertraline and placebo in the treatment of posttraumatic stress disorder. *Archives of General Psychiatry*.

[20] Zohar J, Amital D, Miodownik C (2002). Double-blind placebo-controlled pilot study of sertraline in military veterans with posttraumatic stress disorder. *Journal of Clinical Psychopharmacology*.

[21] Van der Kolk BA, Dreyfuss D, Michaels M (1994). Fluoxetine in posttraumatic stress disorder. *Journal of Clinical Psychiatry*.

[22] Seedat S, Stein DJ, Ziervogel C (2002). Comparison of response to a selective serotonin reuptake inhibitor in children, adolescents, and adults with posttraumatic stress disorder. *Journal of Child and Adolescent Psychopharmacology*.

[23] Schoenfeld FB, Marmar CR, Neylan TC (2004). Current concepts in pharmacotherapy for posttraumatic stress disorder. *Psychiatric Services*.

[24] Cukor J, Olden M, Lee F, Difede J (2010). Evidence-based treatments for PTSD, new directions, and special challenges. *Annals of the New York Academy of Sciences*.

[25] Foa EB, Franklin ME, Moser J (2002). Context in the clinic: how well do cognitive-behavioral therapies and medications work in combination?. *Biological Psychiatry*.

[26] Bschor T, Adli M (2008). Treatment of depressive disorders. *Deutsches Arzteblatt*.

[27] Cascade E, Kalali AH, Kennedy SH (2009). Real-world data on SSRI antidepressant side effects. *Psychiatry*.

[28] Papakostas GI (2010). The efficacy, tolerability, and safety of contemporary antidepressants. *The Journal of Clinical Psychiatry*.

[29] Muench J, Hamer AM (2010). Adverse effects of antipsychotic medications. *American Family Physician*.

[30] Li Y, Xu BY, Xiao F (2009). Effect of modified xiaoyao powder for improving sleep in patients with psychological stress insomnia. *Zhongguo Zhong Xi Yi Jie He Za Zhi*.

[31] Yu HT, Zhu LP, Long B (2006). Clinical observation on treatment of somatic disorder with combination of Xiaoyao Powder and Wendan Decoction. *Chongguo Zhong Xi Yi Jie He Za Zhi*.

[32] Yi ZH, Zhu LP, Long B (2010). Clinical observation on treatment of major depressive disorder by paroxetine combined with chaihu xiaoyao mixture. *Zhongguo Zhong Xi Yi Jie He Za Zhi*.

[33] Yang ZY, Zhang WB, Liu JL (2007). Comparative study of modified Xiaoyao Pill combining amitriptyline on therapeutic effect and compliance in treating patients with depression. *Zhongguo Zhong Xi Yi Jie He Za Zhi*.

[34] Yu GH, Liang SC, Sun QZ (2007). Study on modified Xiaoyao decoction combining Clomipramine treating depression. *Zhongguo Zhong Xi Yi Jie He Za Zhi*.

[35] Luo HC, Qian RQ, Zhao XY (2006). Clinical observation on effect of danzhi xiaoyao powder in treating depression. *Zhongguo Zhong Xi Yi Jie He Za Zhi*.

[36] Qian LQ, Wang B, Niu JY, Gao S, Zhao DY (2010). Assessment of the clinical effect of Chinese medicine therapy combined with psychological intervention for treatment of patients of peri-menopausal syndrome complicated with hyperlipidemia. *Chinese Journal of Integrative Medicine*.

[37] Wei XH, Cheng XM, Shen JS, Wang ZT (2008). Antidepressant effect of Yueju-Wan ethanol extract and its fractions in mice models of despair. *Journal of Ethnopharmacology*.

[38] Zhan CE, Chen JY, Pan F (2004). Effect of modified chaihu shugan powder in treating patients with functional dyspepsia accompanied with depression. *Zhongguo Zhong Xi Yi Jie He Za Zhi*.

[39] Yang YX, Wei PK, Xiu LJ, Zao Y, Shi J, Li YX (2009). Efflect of Bailong Jieyu granules on qulity of life of Patients with cancer-ralated depression. *Chinese Journa1 of Information on TCM*.

[40] Psychiatry Branch of the Chinese Medical Association (2001). *Chinese Classification and Diagnostic Criteria of Mental Disorders Version 3*.

[41] Derogatis LR (1994). *Symptom Checklist-90-R: Administration, Scoring, and Procedures Manual*.

[42] Jin H, Wu WY, Zhang MY (1986). Preliminary analysis of SCL-90 in normal Chinese population. *Chinese Journal of Nervous and Mental Diseases*.

[43] Derogatis LR, Cleary PA (1977). Confirmation of the dimensional structure of the SCL-90: a study in construct validation. *Journal of Clinical Psychology*.

[44] Heller-Boersma JG, Schmidt UH, Edmonds DK (2007). A randomized controlled trial of a cognitive-behavioural group intervention versus waiting-list control for women with uterovaginal agenesis (Mayer-Rokitansky-Küster-Hauser syndrome: MRKH). *Human Reproduction*.

[45] Vaage AB, Thomsen PH, Silove D, Wentzel-Larsen T, Van Ta T, Hauff E (2010). Long-term mental health of Vietnamese refugees in the aftermath of trauma. *British Journal of Psychiatry*.

[46] Wang ZY (1984). The self-report symptom inventory (SCl-90). *Shanghai Archives of Psychiatry*.

[47] Jin S, Yan L, Li B (2010). Quality of life and psychologic distress of recipients after adult living-donor liver transplantation (LDLT)-a study from mainland china. *Transplantation Proceedings*.

[48] Wang X, Gao L, Shinfuku N, Zhang H, Zhao C, Shen Y (2000). Longitudinal study of earthquake-related PTSD in a randomly selected community sample in North China. *American Journal of Psychiatry*.

[49] Linden M, Baumann K, Rotter M, Schippan B (2007). Posttraumatic embitterment disorder in comparison to other mental disorders. *Psychotherapy and Psychosomatics*.

[50] Klarić M, Klarić B, Stevanović A, Grković J, Jonovska S (2007). Psychological consequences of war trauma and postwar social stressors in women in Bosnia and Herzegovina. *Croatian Medical Journal*.

[51] Andreski P, Chilcoat H, Breslau N (1998). Post-traumatic stress disorder and somatization symptoms: a prospective study. *Psychiatry Research*.

[52] Lilly MM, Pole N, Best SR, Metzler T, Marmar CR (2009). Gender and PTSD: what can we learn from female police officers?. *Journal of Anxiety Disorders*.

[53] Van Minnen A, Arntz A, Keijsers GPJ (2002). Prolonged exposure in patients with chronic PTSD: predictors of treatment outcome and dropout. *Behaviour Research and Therapy*.

[54] Spoormaker VI, Van Den Bout J (2005). Depression and anxiety complaints; Relations with sleep disturbances. *European Psychiatry*.

[55] De Leeuw R, Bertoli E, Schmidt JE, Carlson CR (2005). Prevalence of post-traumatic stress disorder symptoms in orofacial pain patients. *Oral Surgery, Oral Medicine, Oral Pathology, Oral Radiology and Endodontology*.

[56] Hong X, Currier GW, Zhao X, Jiang Y, Zhou W, Wei J (2009). Posttraumatic stress disorder in convalescent severe acute respiratory syndrome patients: a 4-year follow-up study. *General Hospital Psychiatry*.

[57] Shipherd JC, Salters-Pedneault K (2008). Attention, memory, intrusive thoughts, and acceptance in PTSD: an update on the empirical literature for clinicians. *Cognitive and Behavioral Practice*.

[58] The Pharmacopoeia Commission of PRC (2005). *Chinese Pharmacopoeia (2005)*.

[59] Zhang Z, Shi Z, Wang L, Liu M (2011). One year later: mental health problems among survivors in hard-hit areas of the Wenchuan earthquake. *Public Health*.

[60] Wang L, Long D, Li Z, Armour C (2011). Posttraumatic stress disorder symptom structure in Chinese adolescents exposed to a deadly earthquake. *Journal of Abnormal Child Psychology*.

[61] Tural U, Onder E, Aker T Effect of depression on recovery from PTSD.

[62] Priebe S, Marchi F, Bini L, Flego M, Costa A, Galeazzi G (2011). Mental disorders, psychological symptoms and quality of life 8 years after an earthquake: findings from a community sample in Italy. *Social Psychiatry and Psychiatric Epidemiology*.

[63] Liu X, Yang Y, Yuan P (2010). A study of the relationship between mental health and menstrual abnormalities in female middle school students from postearthquake Wenchuan. *Bioscience trends*.

[64] Beckham JC, Moore SD, Feldman ME, Hertzberg MA, Kirby AC, Fairbank JA (1998). Health status, somatization, and severity of posttraumatic stress disorder in Vietnam combat veterans with posttraumatic stress disorder. *American Journal of Psychiatry*.

[65] Rauch SAM, Favorite T, Giardino N, Porcari C, Defever E, Liberzon I (2010). Relationship between anxiety, depression, and health satisfaction among veterans with PTSD. *Journal of Affective Disorders*.

[66] Oquendo M, Brent DA, Birmaher B (2005). Posttraumatic stress disorder comorbid with major depression: factors mediating the association with suicidal behavior. *American Journal of Psychiatry*.

[67] Oquendo MA, Friend JM, Halberstam B (2003). Association of comorbid posttraumatic stress disorder and major depression with greater risk for suicidal behavior. *American Journal of Psychiatry*.

[68] Doctor JN, Zoellner LA, Feeny NC (2011). Predictors of health-related quality-of-life utilities among persons with posttraumatic stress disorder. *Psychiatric Services*.

[69] Miller TQ, Smith TW, Turner CW, Guijarro ML, Hallet AJ (1996). A meta-analytic review of research on hostility and physical health. *Psychological bulletin*.

[70] David D, Kutcher GS, Jackson EI, Mellman TA (1999). Psychotic symptoms in combat-related posttraumatic stress disorder. *Journal of Clinical Psychiatry*.

[71] Hamner MB, Frueh BC, Ulmer HG (2000). Psychotic features in chronic posttraumatic stress disorder and schizophrenia: comparative severity. *Journal of Nervous and Mental Disease*.

[72] Connor KM, Hidalgo RB, Crockett B, Malik M, Katz RJ, Davidson JRT (2001). Predictors of treatment response in patients with posttraumatic stress disorder. *Progress in Neuro-Psychopharmacology and Biological Psychiatry*.

[73] Gershuny BS, Baer L, Jenike MA, Minichiello WE, Wilhelm S (2002). Comorbid posttraumatic stress disorder: impact on treatment outcome for obsessive-compulsive disorder. *American Journal of Psychiatry*.

[74] Friedrich WN, Schafer LC (1995). Somatic symptoms in sexually abused children. *Journal of Pediatric Psychology*.

[75] Neylan TC, Marmar CR, Metzler TJ (1998). Sleep disturbances in the Vietnam generation: findings from a nationally representative sample of male Vietnam Veterans. *American Journal of Psychiatry*.

[76] Chilcoat HD, Breslau N (1998). Posttraumatic stress disorder and drug disorders: testing causal pathways. *Archives of General Psychiatry*.

[77] Saladin ME, Brady KT, Dansky BS, Kilpatrick DG (1995). Understanding comorbidity between PTSD and substance use disorders: two preliminary investigations. *Addictive Behaviors*.

[78] Krakow B, Artar A, Warner TD (2000). Sleep disorder, depression, and suicidality in female sexual assault survivors. *Crisis*.

[79] Bao L, Chen J, Huang L (2008). Effects of Xiaoyao Wan on the behavioral despair and stress depression mice. *Zhong Yao Cai*.

[80] Yue GX, Wang ZF, Zhang QL, Zhao X, Yue LF, Ding J (2007). Changes of AMPA receptors and related protein in immobilization stress rats and effect of Xiaoyao Powder. *Journal of Beijing University of Traditional Chinese Medicine*.

[81] Bredt DS, Nicoll RA (2003). AMPA receptor trafficking at excitatory synapses. *Neuron*.

[82] Lu W, Ziff EB (2005). PICK1 interacts with ABP/GRIP to regulate AMPA receptor trafficking. *Neuron*.

[83] Yue GX, Wang ZF, Zhang QL (2007). Changes of central AMPA receptor subunits and related protein mRNA expression in immobilization stressed rats and effect of Xiaoyaosan on them. *Zhongguo Zhong Xi Yi Jie He Za Zhi*.

[84] Xu ZW, Sun Q, Ao HQ, Wang WZ, Fu WJ (2011). Effects of Xiaoyao powder on NR1, NR2A and NR2B mRNA expression in cultured hippocampal neurons of rats under chronic stress. *Journal of Guangzhou University of Traditional Chinese Medicine*.

[85] Xu ZW, Fu WJ, Ao HQ (2009). Effect of Xiaoyaosan on HPA axis negative feedback regulation function of rats with chronic stress. *Journal of Shanxi College of Traditional Chinese Medicine*.

[86] Xu ZW, Ao HQ, Yan C, Wu LL, Wang WZ (2005). Effect of Xiaoyao powder on Ca^2+^ in hippocampal synaptosome of Multi-stress model rats. *Journal of Guangzhou University of Traditional Chinese Medicine*.

[87] Xiu LJ, Wei PK, Liu L (2007). Influence of traditional Chinese recipe Xiaotanjieyu decoction on behavior of mice after chronic swimming stress and on expression of 5-HT1A receptor mRNA in their hippocampus. *Academic Journal of Second Military Medical University*.

[88] Weathers FW, Keane TM, Davidson JRT (2001). Clinician-administered PTSD scale: a review of the first ten years of research. *Depression and Anxiety*.

[89] Connor KM, Davidson JRT (1999). Further psychometric assessment of the TOP-8: a brief interview-based measure of PTSD. *Depression and Anxiety*.

